# Impact of arginine-vasopressin on regional perfusions in a porcine model of post-resuscitation syndrome

**DOI:** 10.1016/j.resplu.2024.100654

**Published:** 2024-05-04

**Authors:** Antoine Bois, Yara Abi Zeid Daou, Naoto Watanabe, Ali Jendoubi, Fanny Lidouren, Estelle Faucher, Nadir Mouri, Bijan Ghaleh, Guillaume Geri, Renaud Tissier, Matthias Kohlhauer

**Affiliations:** a INSERM, U955, IMRB, Team 3, AfterROSC Network, Créteil, France; b Université Univ Paris Est Créteil, INSERM, IMRB, F-94010, Créteil, France; c Ecole Nationale Vétérinaire d’Alfort, IMRB, F-94700, Maisons-Alfort, France; d Assistance Publique-Hôpitaux de Paris, Hôpitaux Universitaires Henri Mondor, Département de Biochimie-pharmacologie, Créteil, France; e Intensive Care Unit, Ambroise Paré Private Hospital, Neuilly-sur-Seine, France

**Keywords:** Cardiac arrest, Resuscitation, Shock, Vasopressin, Vasopressor, Blood flow

## Abstract

**Background:**

Post-cardiac arrest (CA) shock is associated with multiple organ failure, including acute kidney injury, and is the leading cause of early death among patient successfully resuscitated from CA. Arginine-vasopressin (AVP) may be an interesting therapeutic alternative or complement to noradrenaline (NAD) to both control shock and preserve regional, especially renal, organ perfusions.

**Methods:**

18 swine (24–39 kg) were submitted to 14 min of ventricular fibrillation and cardio-pulmonary resuscitation. After return of spontaneous circulation (ROSC), animals randomly received either AVP, NAD or AVP-NAD combination for maintaining a targeted mean arterial pressure of 70 ± 5 mmHg for 6 h. Haemodynamic and biological parameters, including kidney function biomarkers and diuresis, were monitored throughout the follow-up.

**Results:**

Targeted mean arterial pressure was successfully obtained in the NAD (*n* = 6) and the AVP-NAD (*n* = 6) groups, but not in the AVP group (*n* = 6), where 4 animals died. As compared to NAD alone, renal blood flow (2.9 ± 1.15 *vs* 4.36 ± 0.64 mL//kg/min in NAD and AVP-NAD groups) and diuresis were higher in the AVP-NAD group. This was associated with a reduction of carotid blood flow and a more severe metabolic acidosis during the first 3 h of follow-up in the AVP-NAD group as compared to NAD group.

**Conclusion:**

Combination of AVP and NAD improved renal perfusion and diuresis but reduced carotid blood flow as compared to NAD alone in a porcine model of post-resuscitation syndrome. AVP alone failed to manage shock and led to mortality.

## Introduction

Despite improvements in prehospital management and standardization of advanced life support, overall prognosis of cardiac arrest (CA) survivors remains poor.^1^ The course of these patients is usually marked by a post-resuscitation syndrome, which appears during the first 24 h and involves shock and multiple organ failure associated with brain anoxic injury that may progress towards a post-anoxic vegetative state and delayed death.^2^

Early intensive care unit mortality of CA patients is thus mostly related to organ failures within the first 3 days.^3^ Acute circulatory failure, which appears to drive these organs dysfunctions, is frequently observed and may lead to refractory shock.^4^ The post-resuscitation circulatory failure results from the combination of myocardial dysfunction, vasoplegia and hypovolemia.^5^ Underlying mechanisms include brain and myocardial injuries themselves as well as capillary leak syndrome, gut injury leading to bacterial translocation and hormonal defect. Thus, the recommended management of such patients associates intravascular fluids infusion, vasopressor and/or inotrope support.^6^ Based on data in settings like septic shock, noradrenaline (NAD) is the first line recommended vasopressor agent but without clear scientific evidence in the post CA situation. Regards both to the high prevalence and associated prognosis of acute kidney injury in this setting^7^ and to the hormonal defect, arginine-vasopressin (AVP) could be used as preferential vasopressor agent by its action on smooth muscle cell. Very few data have been provided after CA but in other contexts, animal and human studies suggest that AVP could improve renal perfusion and function as compared to NAD.^8^, ^9^, ^10^, ^11^ AVP could then represent an interesting therapeutic alternative to NAD in post-resuscitation syndrome to both control shock and improve renal function.

We hypothesise that AVP infusion could fulfil the vasopressors requirements and preserve regional perfusions, especially renal, as compared to noradrenaline, after CA. The aim of the present study was then to evaluate the effect of AVP, alone or in combination with NAD, as compared to NAD alone on renal and cerebral perfusion in a porcine model of CA.

## Methods

### Ethical considerations

The study protocol was reviewed and approved by the ethical committee ComEth Anses-EnvA-UPEC (Committee No. 16, project 22–105). All experiments were conducted in accordance with the ARRIVE guidelines (Table S1)^12^ and the European Community Standards on the Care and Use of Laboratory Animals.

### Animal preparation

Female swine (race *Large White*), weighing between 24 and 39 kg, were sedated with a mixture of zolazepam and tiletamine (5 mg/kg of each, i.m.) and received an analgesia by methadone (0.75 mg/kg, i.m.). Anaesthesia was then induced and maintained by propofol (bolus of 2 mg/kg followed by continuous i.v. infusion of 10 mg/kg/h). After endotracheal intubation, a conventional mechanical ventilation was applied (tidal volume of 8 mL/kg, respiratory rate of 20 breaths/min, positive end-expiratory pressure of 5 cmH_2_O and inspiratory fraction of oxygen of 30%), continuously adjusted to maintain normocapnia and normoxia. Electrocardiogram, pulse oximetry, blood pressure and rectal temperature were monitored.

Animals were then instrumented with a pressure gauge (Millar®, SPR-524, Houston, TX, USA) positioned by craniotomy in the cerebral cortex to continuously monitor intracranial pressure and two near infrared spectroscopy (NIRS; INVOSTM 5100C, Medtronic®) electrodes were placed on the forehead. Four vascular catheters were inserted using the Seldinger technique, under ultrasound guidance. Two at the right femoral level, one in artery (6 Fr) and one in the vein (9 Fr), enabling the insertion of a pressure gauge (Millar®, SPR-524, Houston, TX, USA) for blood pressure monitoring and the pacemaker probe (see below) respectively. A second arterial catheter fitted with a thermistor was placed in the left femoral artery, used to haemodynamic monitoring with transpulmonary thermodilution principle (PiCCO, Getinge®, Sweden). Finally, a 3-lumen (7 Fr/16 cm) catheter was implanted in the right external jugular vein, for the continuous recording of right atrial pressure and administration of the various drugs. After surgical exposure (*via* median cervicotomy and median laparotomy, respectively), 2.5 mm blood flow probes (PS-Series Probes®, Transonic, NY, USA) were placed around the left internal carotid and left renal arteries. The laparotomy was also used to insert a catheter into the bladder, enabling urine to be collected for sampling and diuresis quantification.

An intravenous infusion of 6 mL/kg/h of Ringer lactate was administered throughout this preparation phase to compensate for sodium and fluid losses associated with anaesthesia and instrumentation. After a period of stabilization, ventricular fibrillation (VF) was induced by a pacemaker positioned in the right ventricle through the femoral venous catheter (A/C 10 V) and left untreated during 14 min (no-flow). During this no-flow period, propofol, crystalloid infusion and mechanical ventilation were discontinued.

### Experimental protocol

After 14 min of no-flow, cardiopulmonary resuscitation (CPR) was initiated using a chest compression system at a rate of 100 compressions/min (LUCAS III, Stryker Medical®, Kalamazoo, MI, USA), and restart of mechanical ventilation. After 3 min of this basic life support, advanced CPR was initiated by administration of adrenaline (0.5 mg, i.v.) every 4 min and electric shocks (8 J/kg) were applied every 2–3 min. An electric shock could be delivered before any adrenaline bolus if coronary perfusion pressure (calculated as the difference between diastolic blood pressure and right atrial pressure) was greater than 30 mmHg.^13^ In case the rhythm became non-shockable, no electric shock was administered. In the absence of success within 15 min, CPR was interrupted, and the animal was not included in the study.

If return of spontaneous circulation (ROSC) was obtained within 15 min, animals were included in the protocol and randomly allocated to receive either AVP, NAD or their combination. Randomization was carried out after CPR, in blocks of three animals (1 animal per group). Treatment administration was then not blinded. Vasopressors were administered as soon as mean arterial pressure dropped below 65 mmHg following ROSC (both vasopressors were started simultaneously in the AVP-NAD group) and delivery rates were continuously adjusted to maintain mean arterial pressure at 70 ± 5 mmHg. AVP was administered at the initial loading dose of 0.4 IU/kg followed by continuous infusion at 0.004 IU/kg/min (Reverpleg®, Opha-Devel Handels, Purkersdorf, Austria) and then modified in steps of 0.004 IU/kg/min up to a maximum dose of 0.05 IU/kg/min (dose retained as there was no therapeutic impact beyond this dose following initial experiments). Similarly, NAD was administered at initial rates of 1 μg/kg/min without loading dose. In the AVP-NAD group, if necessary, AVP administration was preferentially increased up to the maximum rate of 0.05 IU/kg/min before increase in NAD administration rate. In contrary, if mean arterial pressure was higher than 75 mmHg, NAD rate was firstly reduced before AVP until complete weaning of the vasopressors, if possible. During the follow-up, if, mean arterial pressure dropped below 40 mmHg despite vasopressor administration, the animal was considered in refractory shock and euthanized in accordance with our local ethical committee.

**Fig. 1:**
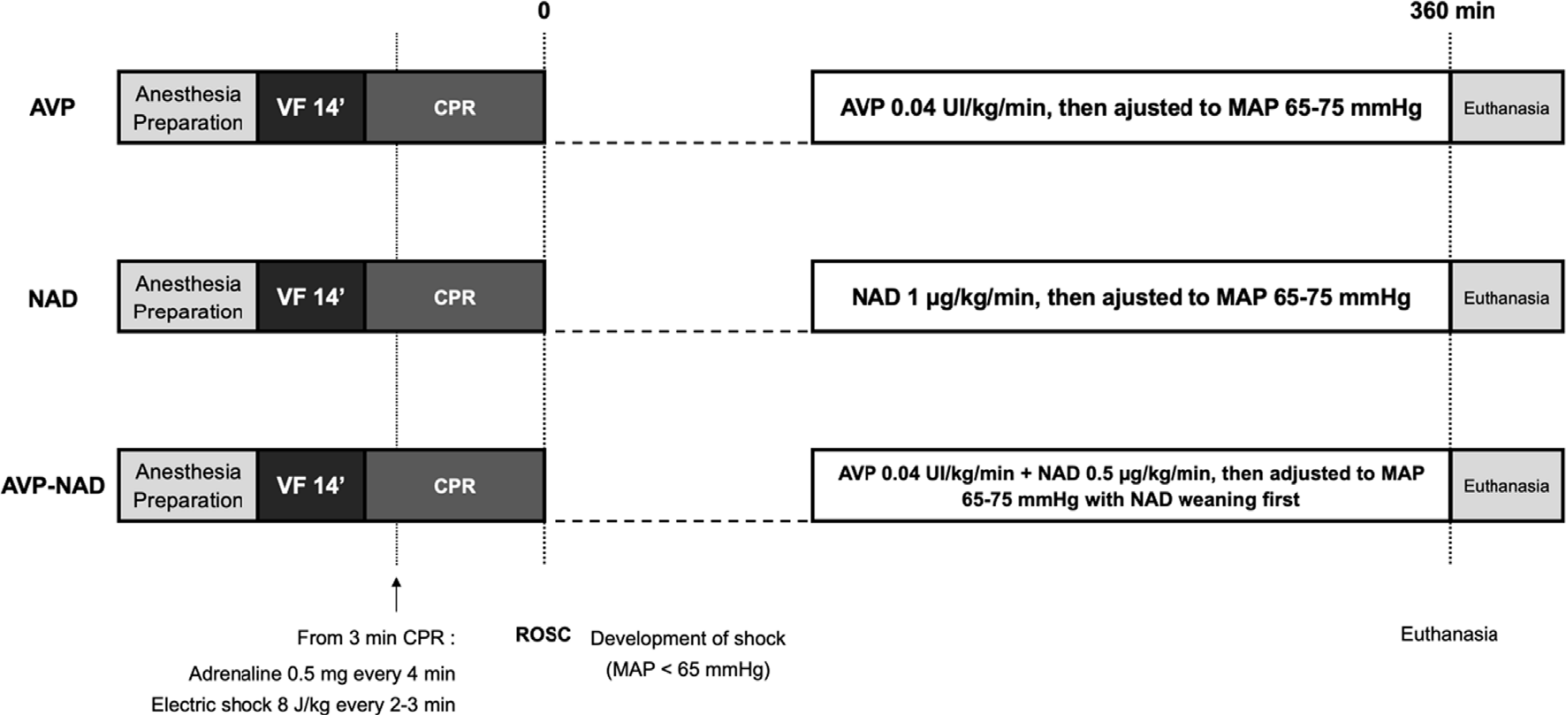
**Experimental study protocol.**AVP, arginine-vasopressin; NAD, noradrenaline; VF, ventricular fibrillation; CPR, cardiopulmonary resuscitation; ROSC, return of spontaneous circulation; MAP, mean arterial pressure.

All surviving swine were monitored for 6 h after ROSC. This period of follow-up was chosen considering the main outcome was the vasopressin effect on renal blood flow (i.e. during shock after ROSC) and the maximum possible duration regards of logistical limitations. At the end of the experiment, all animals were euthanized with an overdose of pentobarbital. The experimental protocol is summarized in Fig. 1.

### Investigated parameters

Heart rate, blood pressure, right atrial pressure, coronary perfusion pressure, intracranial pressure, cerebral perfusion pressure (define as difference between mean arterial pressure and intracranial pressure), and renal and carotid blood flow were recorded continuously using dedicated software (Hem 4.4, Notocord®, France). Respiratory parameters, cerebral oximetry and haemodynamic parameters measured by the PiCCO system were collected hourly. Arterial blood gases, lactate and haemoglobin were measured at baseline and every hour after ROSC. Blood and urinary levels of creatinine and sodium, and blood levels of troponin T, creatine phosphokinase and alanine aminotransferases (ALAT) were measured at baseline and at 180 min and 360 min post-ROSC. The neurofilament light polypeptide (NFL) was also measured at baseline and after 360 min. Urine volume was quantified at the end of the protocol and creatinine clearance was computed as well as fractional excretion of sodium.

### Statistical analysis

Our main study criteria was the improvement of renal blood flow by the association vasopressin and noradrenaline at the end of the follow-up. Based on preliminary data from our lab, we expected a mean value of 3 ± 1 mL/kg/min in the NAD group. In order to detect a clinically relevant improvement of 50% of this value, the number of animal to include were then 6 in each groups with an alpha risk of 5% and beta risk of 20% (unilateral test).

Quantitative parameters were described as mean ± standard error (SEM). Quantitative parameters were compared by analysis of variance for repeated measures, possibly followed by a Fisher LSD test for multiple comparison. A Student *t*-test was performed for parameters with only one measurement. A p-value lower than 0.05 was considered significant. Statistical analysis was performed using GraphPad Prism software (GraphPad Software, California, USA).

## Results

Of the 40 animals used in the study, 22 (55%) were successfully resuscitated but 2 animals did not require any vasopressors administration and 2 animals were resuscitated after 15 min of CPR. Finally, 18 animals were included in the study (6 in each group; Fig. S1). Baseline characteristics were similar across the three groups and summarized in Table 1. CPR characteristics were also similar among the groups, except for a lower low-flow duration for AVP-NAD group (8.0 ± 0.6, 7.5 ± 0.8 and 4.5 ± 0.6 min in AVP, NAD and AVP-NAD groups, respectively, *p* = 0.006). All animals presented a shockable rhythm (ventricular fibrillation) during the CPR. The mean delay before starting vasopressors was 23.8 ± 2.4; 15.3 ± 2.8 and 15.6 ± 3.8 min in AVP, NAD and AVP-NAD groups respectively, *p* = 0.14).

**Table 1:** Baseline characteristics.

**Parameters**	**AVP** **(N = 6)**	**NAD** **(N = 6)**	**AVP-NAD** **(N = 6)**
***Clinical characteristics***			
Weight, kg	32 ± 1	32 ± 2	32 ± 2
Rectal temperature, °C	38.0 ± 0.3	36.8 ± 0.5	37.4 ± 0.3
Respiratory rate, breaths/min	23 ± 1	22 ± 1	25 ± 1
Plateau pressure, cmH_2_O	11 ± 0	12 ± 0	12 ± 0

***CPR characteristics***			
*No-flow*, min	14 ± 0	14 ± 0	14 ± 0
*Low-flow*, min	8.0 ± 0.6 *	7.5 ± 0.8	4.5 ± 0.6 *
Number of electric shock	8 ± 2	5 ± 1	3 ± 1
Number of adrenaline dose	2 ± 0 *	1 ± 0	0 ± 0 *

AVP, arginine-vasopressin; NAD, noradrenaline.*p < 0.05 *versus* NAD group.

**Fig. 2:**
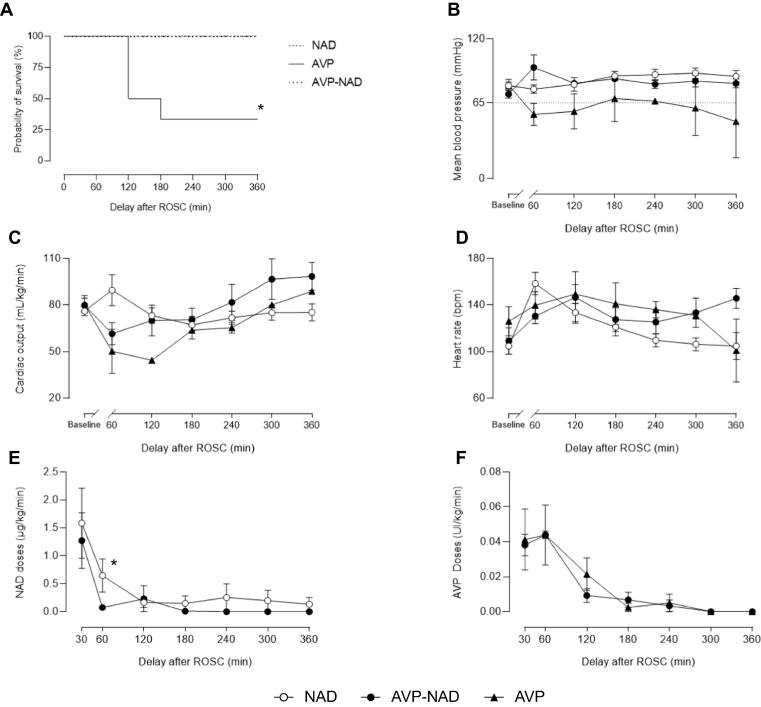
**Management of post-resuscitation shock with NAD, AVP and the AVP-NAD combination represented by the Kaplan-Meier survival curves (A), the mean arterial pressure (B), the cardiac output (C), the heart rate (D), NAD (E) and AVP (F) doses (in the corresponding groups) each hour after ROSC.** AVP, arginine-vasopressin; NAD, noradrenaline; ROSC, return of spontaneous circulation. The baseline time-point corresponds to the value before cardiac arrest. For panel B to F in AVP group: *n* = 6 at 60 min, *n* = 3 at 120 min and *n* = 2 from 180 to 360 min. *n* = 6 at each time point in NAD and AVP-NAD group. *p < 0.05 between groups.

In the AVP group, AVP administration was not effective in maintaining the targeted mean arterial pressure and only 2 animals survived the 6-hours follow-up with low arterial pressure, while shock was adequately controlled for all animals in the two other groups (Fig. 2A and 2B). As illustrated by Fig. 2F, AVP doses were similar between AVP and AVP-NAD groups. Cardiac output value was similar between groups (Fig. 2C and 2D) except at 60 min where NAD had a significantly higher cardiac output than other groups (89.5 ± 9.9 *vs* 61.50 ± 7.1 and 50.2 ± 14.2 ml/min/kg in NAD, AVP-NAD and AVP groups, respectively). As illustrated by Fig. 2E, NAD doses was significantly higher at 60 min after ROSC in NAD group as compared to NAD-AVP group, with 0.65 ± 0.28 µg/kg/min *versus* 0.075 ± 0.03 µg/kg/min, respectively (*p* = 0.034). However, the mean time spent on vasopressors and the mean total dose of NAD received did not significantly differ in the NAD and AVP-NAD groups (185 ± 50 *versus* 149 ± 30 min (*p* = 0.508) and 3606 ± 1832 *vs* 1408 ± 474 μg (*p* = 0.232), respectively). The mean cumulative dose of AVP in the AVP-NAD group was 150 ± 20 IU. A more severe metabolic acidosis related to a higher lactatemia was observed in the AVP-NAD group during the first 3 hours compared to the NAD group (Table 2).

**Table 2:** Regional perfusions parameters and biological results in NAD and AVP-NAD groups.

**Parameters and groups**	**Baseline**	**Delay after ROSC**					
		**60 min**	**120 min**	**180 min**	**240 min**	**300 min**	**360 min**
***Coronary perfusion pressure, mmHg***							
NAD	70 ± 3	68 ± 4	72 ± 6	79 ± 4	80 ± 5	82 ± 5	78 ± 5
AVP-NAD	62 ± 4	84 ± 12	72 ± 2	76 ± 3	71 ± 4	75 ± 6	74 ± 4

***Cerebral perfusion pressure, mmHg***							
NAD	63 ± 4	62 ± 4	65 ± 7	71 ± 4	72 ± 6	73 ± 5	70 ± 5
AVP-NAD	56 ± 4	83 ± 14	68 ± 3	70 ± 4	64 ± 5	68 ± 9	67 ± 5

***Cerebral oximetry by NIRS, %***							
NAD	61 ± 8	54 ± 3	52 ± 2	51 ± 3	52 ± 2	52 ± 3	52 ± 2
AVP-NAD	48 ± 5	46 ± 11	38 ± 8	49 ± 9	52 ± 10	59 ± 9	53 ± 3

***Arterial pH***							
NAD	7.46 ± 0.02	7.33 ± 0.02	7.42 ± 0.01	7.46 ± 0.01	7.46 ± 0.01	7.46 ± 0.01	7.46 ± 0.01
AVP-NAD	7.45 ± 0.01	7.23 ± 0.04 *	7.29 ± 0.02 *	7.36 ± 0.03 *	7.41 ± 0.05	7.42 ± 0.04	7.42 ± 0.04

***PaO_2_, mmHg***							
NAD	166 ± 3	145 ± 6	154 ± 5	158 ± 5	160 ± 4	157 ± 5	158 ± 4
AVP-NAD	156 ± 8	155 ± 7	154 ± 6	157 ± 5	154 ± 5	151 ± 5	150 ± 6

***PaCO_2_, mmHg***							
NAD	38 ± 1	38 ± 1	37 ± 1	36 ± 1	39 ± 1	40 ± 0	39 ± 1
AVP-NAD	39 ± 1	42 ± 3	41 ± 2	38 ± 2	36 ± 3	40 ± 1	40 ± 1

***HCO_3_^–^, mmol/L***							
NAD	26.3 ± 0.8	20.0 ± 0.9	23.6 ± 0.9	24.9 ± 0.9	26.5 ± 1.0	27.6 ± 0.7	27.2 ± 0.5
AVP-NAD	26.7 ± 0.8	17.0 ± 1.1	19.3 ± 1.1 *	21.2 ± 1.2 *	23.7 ± 1.8	25.4 ± 2.1	25.7 ± 2.0

***Arterial lactate, mmol/L***							
NAD	3.1 ± 0.5	9.3 ± 0.7	5.3 ± 0.7	3.0 ± 0.5	2.1 ± 0.4	1.6 ± 0.3	1.4 ± 0.3
AVP-NAD	3.2 ± 0.6	11.9 ± 0.5	10.6 ± 1.0 *	8.1 ± 1.7 *	5.8 ± 1.8 *	4.7 ± 2.0 *	4.4 ± 2.0 *

***Hematocrit, %***							
NAD	31 ± 1	37 ± 2	36 ± 2	34 ± 2	33 ± 2	33 ± 1	32 ± 1
AVP-NAD	28 ± 1 *	32 ± 1 *	32 ± 1 *	31 ± 1	32 ± 1	32 ± 1	32 ± 1

***Natremia, mmol/L***							
NAD	135.3 ± 0.3	134.7 ± 0.6	133.9 ± 0.8	133.6 ± 0.7	134.3 ± 0.6	133.8 ± 0.5	133.7 ± 0.4
AVP-NAD	136.0 ± 0.8	133.5 ± 0.8	133.8 ± 1.1	133.2 ± 1.4	134.1 ± 1.2	134.9 ± 1.2	134.4 ± 1.2

AVP, arginine-vasopressin; NAD, noradrenaline; ROSC, return of spontaneous circulation; NIRS, near-infrared spectroscopy; PaCO_2_, arterial carbon dioxide partial pressure; HCO_3_^–^, bicarbonate.

The baseline time-point corresponds to the value before cardiac arrest.

*p < 0.05 *versus* NAD. N = 6 in each group.

**Fig. 3:**
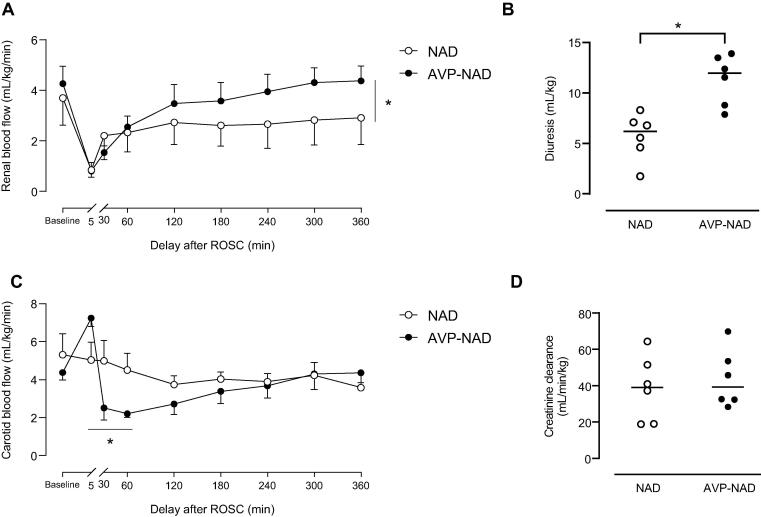
**Regional perfusions and associated parameters in NAD and AVP-NAD groups during the follow-up after ROSC, with renal blood flow (A), the 6-hours diuresis (B), carotid blood flow (C) and creatinine clearance at 360 min after ROSC (D).** AVP, arginine-vasopressin; NAD, noradrenaline; ROSC, return of spontaneous circulation. The baseline time-point corresponds to the value before cardiac arrest. *p < 0.05 between groups. n = 6 in each group.

**Fig. 4:**
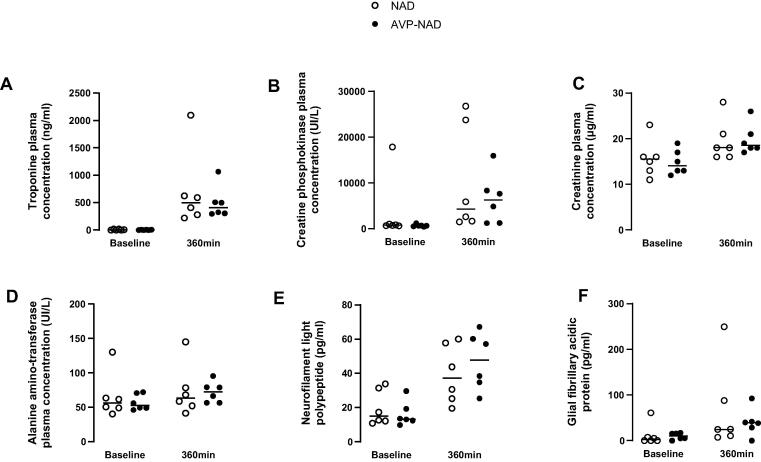
**Biochemical parameters at baseline and 360 min after ROSC in NAD and AVP-NAD groups, including blood levels of troponin T (A), creatine phosphokinase (B), creatinine (C), alanine amino-transferase (D), neurofilament light polypeptide (E) and glial fibrillary acidic protein (F).** AVP, arginine-vasopressin; NAD, noradrenaline; ROSC, return of spontaneous circulation; Tn T, troponin T; CPK, creatine phosphokinase; ALAT, alanine aminotransferase; NFL, neurofilament light polypeptide; GFAP, glial fibrillary acidic protein. The baseline time-point corresponds to the value before cardiac arrest. *n* = 6 in each group.

Due to the absence of control of shock in the AVP group, all other results were only compared between NAD and AVP-NAD groups (Fig. 3, Fig. 4).

After ROSC, the association of AVP and NAD allowed a significant better recovery of the initial drop in renal blood flow as compared to NAD only (Fig. 3A). As example, the renal blood flow after 6 h following ROSC was 2.9 ± 1.15 *vs* 4.36 ± 0.64 mL/min/kg in NAD and AVP-NAD groups, respectively. The total volume of urine produced over 6 h was also significantly greater in the AVP-NAD group (11.9 ± 3.0 *vs* 5.7 ± 1.0 mL/kg, *p* = 0.002; Fig. 3B). However, despite having better kidney perfusion and urinary output, creatinine clearance and fractional excretion of sodium did not differ between groups at 360 min post-ROSC (39.05 *versus* 39.21 mL/min/kg (*p* = 0.612) and 0.74 *vs* 1.09% (*p* = 0.292) in the NAD and AVP-NAD groups, respectively).

The carotid blood flow was maintained after the ROSC and was significantly higher at 5 min post-ROSC in the AVP-NAD group compared with NAD group (this difference preceding the introduction of any vasopressor). During the first hour of follow-up, carotid blood flow was reduced in the AVP-NAD group (Fig. 3C). The cerebral perfusion pressure, tissue cerebral oximetry (NIRS) and NFL and GFAP blood levels did not differ between both groups (Table 2, Fig. 4).

Other markers of regional perfusions, as coronary perfusion pressure, troponin blood levels, alanine aminotransferase blood levels and creatine phosphokinase were similar between the groups (Fig. 4).

## Discussion

Here, we described the effect of AVP alone or in combination with NAD to control shock after CA and its impact on regional perfusions in a porcine model of post-resuscitation syndrome. To our knowledge, this is the first experimental study to examine in detail the effect of AVP in this setting.

In our study, AVP alone failed to control arterial pressure, leading to higher mortality. This result differs from other experimental porcine models, as endotoxic or haemorrhagic shock,^14^, ^15^, ^16^, ^17^ where AVP was quite effective to manage shock. Nonetheless, in a porcine model of cardiogenic shock, Müller *et al.* reported that AVP was associated with a decrease in cardiac output as well as coronary, cerebral or renal perfusion rates.^18^ This may explain why AVP alone failed to manage the cardiogenic component in our model of post-resuscitation shock. Indeed, post-resuscitation shock includes a component of myocardial stunning, well described in the literature and secondary to ischemia–reperfusion,^5^ repeated defibrillation^19^ or the toxicity of adrenaline,^20^ among other factors. Accordingly, cardiac output was higher at the first hour in the NAD group as compared to AVP and AVP-NAD groups. This could suggest that AVP lacks the inotropic effect needed to counteract myocardial stunning, or even possess a negative inotropic effect at high doses,^21^ as compared to NAD that could be a good therapeutic option in cardiogenic failure.^22^ Recently, some data suggests that AVP may contribute to microvascular dysfunction after an acute coronary syndrome supporting this hypothesis.^23^ In AVP-NAD group, coronary perfusion pressure was equivalent to that of the NAD group. However, this does not rule out a difference in coronary flow, which was described as a potential causal factor for the fall in cardiac output in the previously mentioned study.^18^ Another explanation to the failure of AVP alone in our study could be an interspecies difference in pharmacokinetics. Swine naturally produce lysine-vasopressin and not arginine-vasopressin,^24^ which may explain a slower correction of mean arterial pressure and therefore, in part, greater severity. However, we cannot exclude that, in our study, AVP group presented a significantly longer low-flow duration. This high variability between groups could be responsible for the absence of efficacy of AVP alone in controlling shock after ROSC.

In the AVP-NAD and NAD groups, blood pressure was controlled adequately. It is consistent with the only human clinical study to investigate the use of AVP in post-resuscitation syndrome, which reported that advanced circulatory failure refractory to standard therapies could be corrected by the addition of AVP to NAD.^25^ In our model, the AVP-NAD group showed lower NAD consumption than the NAD-only group. Although not statistically significant in the present work, this result is consistent with studies in sepsis, where AVP decrease catecholamine needs.^26^ The vasopressor effect of AVP thus appears to be preserved in swine with post-resuscitation shock.

Regarding the regional perfusion, the association AVP-NAD improved renal parameters after CA in our study as compared to NAD alone. Renal blood flow was greater during the 6-hours follow-up and diuresis more than doubled even if creatinine clearance failed to reach statistical significance, possibly due to the early time-point of the analysis. This is consistent with data in the experimental literature from several animal models of endotoxin or septic shock, or even haemorrhage, which show an improvement in renal blood flow^8^, ^17^ or an improvement in renal function under AVP.^9^, ^27^ Here, this effect may have been enhanced by the very early initiation of AVP which is associated with its benefit on renal function in sepsis.^26^ A difference in fluid infusion could also be discussed. Although no fluid infusion was administered after ROSC, we note a difference in hemodilution profiting the AVP-NAD group during the first 2 h of follow-up (possibly related to the AVP infusion directly). However, this difference between the groups already existed at baseline and does not seem important enough to explain such an effect on diuresis. In humans, AVP was associated with a reduction in the number of cases of acute kidney injury and use of renal replacement therapy during septic shock.^10^, ^11^ Thus, our results seem encouraging for improving renal function and associated outcome after CA.

We also found a transient reduction in carotid blood flow during the first part of follow-up under AVP-NAD compared with NAD alone. The optimal carotid blood flow is yet unknown, and current guidelines recommend only that blood pressure be adjusted to maintain optimal cerebral perfusion pressure.^6^ However, a reduction in cerebral “hyperaemia” following the ROSC may allow cerebral blood flow to be better adapted to metabolic needs, according to some literature.^28^ Moreover, this lower carotid blood flow under AVP did not appear to be harmful to brain function, since cerebral perfusion pressure, cerebral tissue oximetry, and NFL and GFAP blood levels were similar between the two groups during follow-up. Further studies are therefore needed to determine whether AVP has a positive or negative effect on cerebral circulation after CA.

Finally, there was a more severe lactic acidosis in AVP-NAD group compared with NAD group despite similar blood pressure control. This observation may suggest a more frequent occurrence of mesenteric or peripheral ischemia with AVP. Impaired superior mesenteric and cutaneous perfusion under AVP has already been reported.^14^

Our study presents some limitations. First, as our model is severe, it is marked by a significant loss of subjects before randomization. For this reason, the number of animals included is small and our study undoubtedly suffers from a lack of statistical power. Second, post-resuscitation shock was rapidly controlled as shown by the rapid weaning of vasopressors. It is possibly related to the fact that swine are young and healthy, without known underlying cardiac disease, in contrast to human clinical reality, where dependence on vasopressor agents is often more prolonged due to associated comorbidities (notably cardiac). Third, the dose of AVP to be used as a vasopressor agent in swine is not standardized and varies widely in the literature depending on the model studied (as summarized in supplemental Table S2). The dose chosen in our study is high and may have caused adverse events as discussed above. Coronary and mesenteric blood flow monitoring could have been interesting to discuss the effects of this vasopressor but would have made an already long instrumentation period even worse. Lastly, a difference in low-flow duration between the groups should be noted, leading to a cautious interpretation of the results.

## Conclusion

AVP alone failed to manage shock in a porcine model of post-resuscitation syndrome, leading to a high mortality. In combination to NAD, AVP may be enable regional perfusions to be redistributed with and improvement of renal blood flow and a possible limitation of carotid blood flow. By this increase in renal blood flow, association of AVP with NAD improved diuresis but not creatine clearance. These data may lead the way to future investigations to optimize AVP management in post-resuscitation syndrome in order to obtain renal benefit with fewer deleterious effects.

Furthermore, other experimental studies comparing the combination of AVP with an inotrope agent like dobutamine to NAD alone could help to clarify the cardiogenic effect of AVP in post-resuscitation syndrome.

## CRediT authorship contribution statement

**Antoine Bois:** Writing – review & editing, Writing – original draft, Methodology, Investigation, Formal analysis, Data curation, Conceptualization. **Yara Abi Zeid Daou:** Investigation. **Naoto Watanabe:** Investigation. **Ali Jendoubi:** Investigation. **Fanny Lidouren:** Methodology, Investigation. **Estelle Faucher:** Investigation. **Nadir Mouri:** Investigation. **Bijan Ghaleh:** Supervision, Resources, Project administration. **Guillaume Geri:** Writing – review & editing, Methodology, Conceptualization. **Renaud Tissier:** Writing – review & editing, Validation, Supervision, Resources, Project administration, Methodology, Formal analysis, Conceptualization. **Matthias Kohlhauer:** Writing – review & editing, Visualization, Validation, Supervision, Resources, Project administration, Methodology, Investigation, Formal analysis, Conceptualization.

## Declaration of competing interest

The authors declare the following financial interests/personal relationships which may be considered as potential competing interests: R Tissier and M Kohlhauer are shareholders of a start-up company dedicated to total liquid ventilation (Orixha).
