# Supplementary material for: Impact of arginine-vasopressin on regional perfusions in a porcine model of post-resuscitation syndrome
Source: Resusc Plus. 2024 May 4;18:100654. doi: 10.1016/j.resplu.2024.100654 (PMC11074971; doi:10.1016/j.resplu.2024.100654)
Supplement: Supplementary Data 1 [file mmc1.docx]

**SUPPLEMENTAL DATA**

**Table S1. ARRIVE guidelines checklist**

**AVP, arginine-vasopressin ; NAD, noradrenaline ; CA, cardiac arrest**

|  | **Item** | **Recommendation** |
| --- | --- | --- |
| **TITLE** | **1** | Impact of arginine-vasopressin on regional perfusions in a porcine model of post-resuscitation syndrome |
| **ABSTRACT** | **2** | AVP may be an alternative to NAD to control shock after CA and improve outcome.  18 swine were submitted to 14 min of ventricular fibrillation and cardio-pulmonary resus-citation. After return of spontaneous circulation (ROSC), animals randomly received either AVP, NAD or AVP-NAD combination for maintaining a targeted mean arterial pressure (MAP) of 70 ± 5 mmHg for 6 hours. Combination of AVP and NAD improved renal perfusion and diuresis but reduced carotid blood flow as compared to NAD alone in a porcine model of post-resuscitation syndrome. AVP alone failed to manage shock and led to mortality. |
| **INTRODUCTION** |  |  |
| - **Background** | **3** | Post-cardiac arrest (CA) shock is associated with multiple organ failure, including acute kidney injury, and is the leading cause of early death among patient successfully resuscitated from CA. Arginine-vasopressin (AVP) may be an interesting therapeutic alternative or complement to noradrenaline (NAD) to both control shock and preserve regional, especially renal, organ perfusions. |
| - **Objectives** | **4** | The aim of the present study was then to evaluate the effect of AVP, alone or in combination with NAD, as compared to NAD alone on renal and cerebral perfusion in a porcine model of CA. |
| **METHODS** |  |  |
| - **Ethical statement** | **5** | The study protocol was reviewed and approved by the ethical committee ComEth Anses-EnvA-UPEC (Committee No. 16, project 22-105). |
| - **Study design** | **6** | It’s an experimental study in open-label. Succesfully resuscitated swine presenting a shock define as mean arterial pressure lower than 65 mmHg were included and randomly allocated to receive either AVP, NAD or their combination  Pigs were not included if we failed to obtain a return of spontaneous circulation, if we successfully resuscitated them after 15 min of CPR or if they did not develop shock.  There were no exclusion criteria. |
| - **Experimental procedures** |  | The swines were sedated with a mixture of zolazepam and tiletamine (5 mg/kg of each, i.m.) and received an analgesia by methadone (0.75 mg/kg, i.m.). Anaesthesia was then induced and maintained by propofol (bolus of 2 mg/kg followed by continuous i.v. infusion of 10 mg/kg/h). After endotracheal intubation, a conventional mechanical ventilation was applied (tidal volume of 8 mL/kg, respiratory rate of 20 breaths/min, positive end-expiratory pressure of 5 cmH_2_O and inspiratory fraction of oxygen of 30%), continuously adjusted to maintain normocapnia and normoxia. Electrocardiogram, pulse oximetry, blood pressure and rectal temperature were monitored.  Animals were then instrumented with a pressure gauge (Millar®, SPR-524, Houston, TX, USA) positioned by craniotomy in the cerebral cortex to continuously monitor intracranial pressure (ICP) and two near infrared spectroscopy (NIRS; INVOSTM 5100C, Medtronic®) electrodes were placed on the forehead. Four vascular catheters were inserted using the Seldinger technique, under ultrasound guidance. Two at the right femoral level, one in artery (6 Fr) and one in the vein (9 Fr), enabling the insertion of a pressure gauge (Millar®, SPR-524, Houston, TX, USA) for blood pressure monitoring and the pacemaker probe (see below) respectively. A second arterial catheter fitted with a thermistor was placed in the left femoral artery, used to hemodynamic monitoring with transpulmonary thermodilution principle (PiCCO, Getinge®, Sweden). Finally, a 3-lumen (7 Fr/16 cm) catheter was implanted in the right external jugular vein, for the continuous recording of right atrial pressure and administration of the various drugs. After surgical exposure (*via* median cervicotomy and median laparotomy, respectively), 2.5 mm blood flow probes (PS-Series Probes®, Transonic, NY, USA) were placed around the left internal carotid and left renal arteries. The laparotomy was also used to insert a catheter into the bladder, enabling urine to be collected for sampling and diuresis quantification.  An intravenous infusion of 6 mL/kg/h of Ringer lactate was administered throughout this prepara-tion phase to compensate for sodium and fluid losses associated with anaesthesia and instrumentation. After a period of stabilization, ventricular fibrillation (VF) was induced by a pacemaker posi-tioned in the right ventricle through the femoral venous catheter (A/C 10 V) and left untreated during 14 min (no-flow). During this no-flow period, propofol, crystalloid infusion and mechanical ventilation were discontinued |
| - **Experimental animals** |  | Study included female swine (hybrid race *Large White* – *Landrace*), weighting between 24 and 39 kg. Before experiments, we took care of the animals in accordance to European Community Standards on the Care and Use of Laboratory Animals.  Follow-up took 6 hours, during which time swine were sedated. A the end of the experiment, the animals were euthanized with a letal dose of pentobarbital. |
| - **Housing and husbandry** |  | The cages were located inside the laboratory. Swines arrives at least 5 days before the planned experiment. They had always water available and were fed at least twice a day. The day/night rhythm was maintained by automatic lighting with a timer. |
| - **Sample size** | **10** | A number of 6 animals per group will be required to show a 50% increase in renal perfusion rate (risk β = 10%). Considering animals that did not meet the inclusion criteria (failed resuscitation, no need for vasopressor), 40 animals were used for the study, with 18 included. |
| - **Allocating animals to experimental groups** | **11** | The animals were randomized in blocks of 3, i.e. we drew lots to allocate the groups of three pigs to be included, with one pig per group. |
| - **Experimental outcomes** | **12** | The main outcome was renal blood flow under AVP or AVP-NAD as compared to NAD alone for similar shock control. Secondary outcomes included carotid blood flow, diuresis and biological parameters. |
| - **Statistical methods** | **13** | Quantitative parameters were described as mean ± standard error (SEM) when normally distribut-ed or by median [95%CI] otherwise. Quantitative parameters were compared by analysis of vari-ance for repeated measures, possibly followed by a Fisher LSD test for multiple comparison. A Student t-test was performed for parameters with only one measurement. A p-value lower than 0.05 was considered significant. Statistical analysis was performed using GraphPad Prism soft-ware (GraphPad Sotwear, California, USA). |
| **RESULTS** |  |  |
| - **Baseline data** | **14** | Baseline characteristics were similar across the three groups, with a mean weight of 32 kg and a mean rectal temperature of 37,4 °C.  Cardiopulmonary resuscitation characteristics (number of electric shock and dose of adrenaline) were also similar across the groups, except for a trend in low-flow duration (8.0, 7.5 and 4.5 min in AVP, NAD and AVP-NAD groups, respectively, p = 0.006). |
| - **Numbers analyzed** | **15** | Of the 40 animals used in the study, 22 were successfully resuscitated but 2 animals did not require any vasopressors administration and 2 animals were resuscitated after 15 min of CPR. Finally, 18 animals were included in the study :6 in each group. To compare the control of shock (blood presssure, cardiac output), we compare the three groups and thus analyed all included animals. Considering, regional perfusions we only compared AVP-NAD and NAD only groups (because unterpretable in the AVP only group due to the absence of shock control) |
| - **Outcomes and estimation** | **16** | - In the AVP group, AVP administration was not effective in maintaining the targeted MAP and only 2 animals survived the 6-hours follow-up, while shock was adequately controlled for all animals in the two other groups - The mean time spent on vasopressors and the mean total dose of NAD received did not significantly differ in the NAD and AVP-NAD groups (185 ± 50 *versus* 149 ± 30 min (p = 0,508) and 3606 ± 1832 *vs* 1408 ± 474 μg (p = 0,232), respectively) - Associtation of AVP and NAD allowed a significant better recovery of the initial drop in renal blood flow as compared to NAD only. As example, the renal blood flow after 6 hours following ROSC was 2.9 ± 1.15 *vs* 4.36 ± 0.64 mL/min/kg in NAD and AVP-NAD groups, respectively. - The total volume of urine produced over 6 hours was also significantly greater in the AVP-NAD group (11.9 ± 3.0 *vs* 5.7 ± 1.0 mL/kg, p = 0.002). - Creatinine clearance and fractional excretion of sodium didn’t differ between groups at 360 min post-ROSC (33.7 *versus* 46.7 mL/min (p = 0.320) and 0.74 *vs* 1.09% (p = 0.292) in the NAD and AVP-NAD groups, respectively) - During the first hour of follow-up, carotid blood flow was reduced in the AVP-NAD group. - The cerebral perfusion pressure, tissular cerebral oximetry (NIRS) and NFL and GFAP blood levels did not differ between both groups. |
| - **Adverse events** | **17** | - A more severe metabolic acidosis related to a higher lactatemia was observed in the AVP-NAD group during the first three hours compared to the NAD group (e.g. at 120 min after ROSC, pH was 7.29 ± 0.02 vs 7.42 ± 0.01 in AVP-NAD and NAD, respectively). - AVP alone led to high mortality |
| **DISCUSSION** |  |  |
| - **Interpretation/scientific implications** | **18** | - AVP alone failed to manage post-resuscitation shock, unlike other types of shock, possibly due to failure to manage the cardiogenic component - The combination AVP-NAD provides good control of blood pressure, as compared to NAD alone (the standard of care) - These association was associated with a higher renal blood flow and diuresis, which is consistent with experimental and clinical human data in other situations. - This was also associated with a reduction of carotid blood flow as compared to NAD alone. The impact of AVP on cerebral perfusion is yet unknow. - Our study as some limitations : First, as our model is severe, it is marked by a significant loss of subjects before randomization. For this reason, the number of animals included is small and our study undoubtedly suffers from a lack of statistical power. Second, post-resuscitation shock is rapidly controlled as shown by the rapid weaning of vasopressors. It is possibly related to the fact that swine are young and healthy, without known underlying cardiac disease, in contrast to human clinical reality, where dependence on vasopressor agents is often more prolonged due to associated comorbidities (notably cardiac). Third, the dose of AVP to be used as a vasopressor agent in swine is not standardized and varies widely in the literature depending on the model studied (as summarized in supplemental table S2). The dose chosen in our study is high and may have caused adverse events as discussed above. Coronary and mesenteric blood flow monitoring could have been interesting to discuss the effects of this vasopressor but would have made an already long instrumentation period even worse. Lastly, a difference in low-flow duration between the groups should be noted, leading to a cautious interpretation of the results. |
| - **Generalisability/translation** | **19** | The impact of AVP on the kidney perfusion after cardiac arrest is interesting considering the high frequency of acute kidney injury and the poor outcome associated with in the post-resuscitation syndrome  Further studies are needed to eliminate the absence of deleterious effect on other organs, especially cerebral or mesenteric perfusions. |
| - **Funding** | **20** | None |

**Figure S1. Flowchart of the study**

CPR, cardiopulmonary resuscitation ; AVP, arginine-vasopressine ; NAD, noradrenaline

**Table S2. Bibliographic review of AVP management when used alone in different porcine models of shock.**

AVP, arginine-vasopressine ; NA, non-available ; CA, cardiac arrest ; ECPR : extracorporeal cardiopulmonary resuscitation

| Reference | Model | AVP dose (IU/kg/min) | Loading dose (UI/kg) | Proprietary medicinal |
| --- | --- | --- | --- | --- |
| Voelckel *et al.*, 2003 ^1^ | Hemorrhagic shock after liver trauma | 0,04 | Yes (0,4) | NA |
| Martikainen *et al.*, 2003 ^2^ | Endotoxin shock | 0,001 | No | Pitressin® |
| Stadbauer *et al.*, 2007 ^3^ | Hemorrhagic shock after abdominal  vascular injury | 0,08 | Yes (0,4) | Pitressin® |
| Hiltebrand *et al.*, 2007 ^4^ | Septic shock by fecal peritonitis | 0,06 | No | POR-8® |
| Müller *et al.*, 2008 ^5^ | Transient myocardial ischemia/reperfusion (without CA) | 0,005 | No | NA |
| Simon *et al.*, 2009 ^6^ | Septic shock by fecal peritonitis | 0,0003 | No | NA |
| Bomberg *et al.*, 2013 ^7^ | Cardiopulmonary bypass (without CA) | 0,006 | No | NA |
| Gazmuri *et al.*, 2017 ^8^ | Hemorrhagic shock after liver trauma | 0,04 | No | Pitressin® |
| Klein *et al.*, 2021^9^ | Post-resuscitation (cardiogenic) shock with ECPR after coronary occlusion | 0,0001 | No | Reverpleg® |

**References**

1. Voelckel WG, Raedler C, Wenzel V, et al. Arginine vasopressin, but not epinephrine, improves survival in uncontrolled hemorrhagic shock after liver trauma in pigs*: *Crit Care Med* 2003; 31: 1160–1165.

2. Martikainen TJ, Tenhunen JJ, Uusaro A, et al. The Effects of Vasopressin on Systemic and Splanchnic Hemodynamics and Metabolism in Endotoxin Shock: *Anesth Analg* 2003; 97: 1756–1763.

3. Stadlbauer KH, Wagner-Berger HG, Krismer AC, et al. Vasopressin improves survival in a porcine model of abdominal vascular injury. *Crit Care* 2007; 11: R81.

4. Hiltebrand LB, Krejci V, Jakob SM, et al. Effects of Vasopressin on Microcirculatory Blood Flow in the Gastrointestinal Tract in Anesthetized Pigs in Septic Shock. *Anesthesiology* 2007; 106: 1156–1167.

5. Müller S, How O-J, Hermansen S, et al. Vasopressin impairs brain, heart and kidney perfusion: an experimental study in pigs after transient myocardial ischemia. *Crit Care* 2008; 12: R20.

6. Simon F, Giudici R, Scheuerle A, et al. Comparison of cardiac, hepatic, and renal effects of arginine vasopressin and noradrenaline during porcine fecal peritonitis: a randomized controlled trial. *Crit Care* 2009; 13: R113.

7. Bomberg H, Bierbach B, Flache S, et al. Endothelin and vasopressin influence splanchnic blood flow distribution during and after cardiopulmonary bypass. *J Thorac Cardiovasc Surg* 2013; 145: 539–547.

8. Gazmuri RJ, Whitehouse K, Whittinghill K, et al. Early and sustained vasopressin infusion augments the hemodynamic efficacy of restrictive fluid resuscitation and improves survival in a liver laceration model of hemorrhagic shock. *J Trauma Acute Care Surg* 2017; 82: 317–327.

9. Klein T, Grandmougin D, Liu Y, et al. Comparison of Vasopressin versus Norepinephrine in a Pig Model of Refractory Cardiogenic Shock Complicated by Cardiac Arrest and Resuscitated with Veno-Arterial ECMO. *Shock*; Publish Ahead of Print. Epub ahead of print 3 February 2021. DOI: 10.1097/SHK.0000000000001747.
